# Comparison of Ropivacaine versus Bupivacaine in Spinal-Induced Hypotension in Preeclampsia Patients: A Randomized Control Trial

**DOI:** 10.5812/aapm-142646

**Published:** 2024-02-29

**Authors:** Morteza Hashemian, Mohsen Barouni, Zahra Honarvar, Katayoun Alidousti, Seyed Amir Mohajerani, Leila Rezaeizadeh

**Affiliations:** 1 Department of Anesthesiology and Pain Medicine, Kerman University of Medical Sciences, Kerman, Iran; 2 Research Center for Health Services Management, Institute for Future Studies in Health, Kerman, Iran; 3 Department of Obstetrics and Gynecology, Kerman University of Medical Sciences, Kerman, Iran; 4 Department of Midwifery, Kerman University of Medical Sciences, Kerman, Iran; 5 Department of Anesthesiology and Pain Medicine, Shahid Beheshti University of Medical Sciences, Tehran, Iran

**Keywords:** Block, Bupivacaine, Hypotension, Preeclampsia, Ropivacaine, Spinal

## Abstract

**Background:**

Spinal anesthesia is considered to be the safest method of anesthesia for cesarean sections in patients with preeclampsia. Patients with preeclampsia are at an increased risk of experiencing severe hypotension following spinal anesthesia, which could have more profound and deleterious effects on both the fetus and the mother. However, bupivacaine, the most commonly used drug, can induce severe hypotension even at low doses. The purpose of this study is to minimize post-spinal hypotension in both the mother and the fetus.

**Objectives:**

To determine and compare the reduction in hypotension following spinal anesthesia in patients with preeclampsia between the ropivacaine and bupivacaine groups.

**Methods:**

In a randomized clinical trial, a total of 90 parturients with preeclampsia undergoing spinal anesthesia were enrolled and randomly divided into 2 groups: One receiving ropivacaine and the other receiving bupivacaine. The dose of spinal ropivacaine was 15 mg of a 0.5% solution, and the dose of bupivacaine was also 15 mg of a 0.5 % solution. Hemodynamic parameters, including systolic and diastolic blood pressure and heart rate, were recorded following the administration of spinal anesthesia. Pain scores and the time until the return of motor movement were also documented.

**Results:**

For statistical analysis, the *t*-test, Chi-square, and ANOVA tests were utilized to compare the groups. Demographic variables, including maternal age, gestational age, parity, and gravidity, were not significantly different between the 2 groups. The trend of mean systolic blood pressure (SBP) was significantly lower in the bupivacaine group compared to the ropivacaine group at all measured time points in the study (P < 0.05). The amount of ephedrine used after spinal anesthesia was significantly different at 2 and 4 minutes in the ropivacaine group compared to the bupivacaine group (P = 0.012, P = 0.025). Post-operative pain scores at 1 hour in recovery were not significantly different between the ropivacaine and bupivacaine groups (P = 0.015). The time to knee movement was also significantly shorter in the ropivacaine group compared to the bupivacaine group (P < 0.001).

**Conclusions:**

Ropivacaine reduces the incidence of hypotension in spinal anesthesia compared to bupivacaine for cesarean section in patients with preeclampsia. This is attributed to a lower occurrence of spinal-induced hypotension, improved hemodynamic control, reduced ephedrine usage, and faster patient ambulation. A future study could focus on investigating different dosages of both drugs with a larger number of participants.

## 1. Background

Preeclampsia is characterized by hypertension, associated with mediated endothelial injury that can result in proteinuria and kidney damage. Preeclampsia stands as one of the most common causes of mortality or morbidity in pregnancy, with various side effects requiring delivery as soon as possible (1). Spinal anesthesia is considered the safest method of anesthesia for cesarean sections in patients with preeclampsia, given the challenges associated with airway management in pregnant women. Additionally, parturients experience a hypercoagulable state, making early ambulation crucial. This emphasizes the advantages of spinal anesthesia, particularly with a drug that provides a prolonged sensory block and a shorter motor block. However, the proper level of anesthesia for cesarean section is T4, which is associated with an increased risk of sympathetic block and subsequent hemodynamic instability. Hypotension and bradycardia are the most common side effects of spinal anesthesia, occurring in up to 64% - 100% of pregnant women undergoing cesarean delivery (2). Although the mechanism of hypertension in preeclamptic patients is associated with renin-angiotensin, all hypertensive patients, including those with preeclampsia, are at an increased risk of hypotension after spinal anesthesia due to lower intravascular volume (3), which could be more profound and more deleterious for the fetus and mother due to a greater decrease in mean arterial blood pressure (MAP) and subsequent cerebral blood flow (CBF) and the risk of intracranial hemorrhage (ICH) (4).

Bupivacaine is the most common analgesic used in spinal anesthesia for cesarean section (5); however, bupivacaine has the side effect of a significant decrease in systolic blood pressure (SBP). A lower dose of bupivacaine (8 - 12 mg) is commonly used for spinal anesthesia; however, it is associated with a high incidence of hypotension and complications for both mother and fetus (6).

The rationale for comparing ropivacaine versus bupivacaine is grounded in the availability of these 2 drugs in the market, cost-effectiveness, and comparable dosages. Additionally, ropivacaine is a long-acting amide local anesthetic with structural and pharmacodynamic similarities to bupivacaine. Ropivacaine is less cardiotoxic and CNS-toxic compared to bupivacaine. Ropivacaine at a dose of 10 - 25 mg is proposed for spinal anesthesia in cesarean delivery due to the advantages of a lower incidence of hypotension and a shorter duration of motor block (7, 8). A previous report showed that hyperbaric ropivacaine provided a similar spinal anesthesia effect with a shorter duration of sensory and motor block compared to hyperbaric bupivacaine for cesarean delivery (9). Some researchers postulate that ropivacaine may be a better replacement for bupivacaine owing to a better separation between the motor and sensory blockade than bupivacaine (10). It is presumed that ropivacaine has a significantly higher selectivity for sensory fibers than for motor and autonomic fibers due to its lower lipophilic capacity compared with bupivacaine (11). In this study, we hypothesize that the decrease in blood pressure after spinal anesthesia in preeclampsia patients is less with ropivacaine compared to bupivacaine.

## 2. Objectives

To compare the incidence of a decrease in hypotension after spinal anesthesia in preeclampsia patients between the ropivacaine and bupivacaine groups.

## 3. Methods

### 3.1. Ethics and Participants

This was a prospective, double-blind, randomized clinical trial approved by the National Registering Clinical Trials with No: IRCT20150317021497N6 and University Ethics Committee No: IRB.KMU.AH.REC.1398.151. A total of 90 patients with preeclampsia, aged 22 - 40 years, and with full-term (> 37 weeks of gestation), ASA class 2 (hypertension), and elective cesarean delivery were enrolled in the study and divided into 2 groups.

After providing signed informed consent, the women were randomly allocated to 1 of 2 groups. Randomization was conducted using a computerized program in which patient identification codes were entered, and a random number was assigned to each patient. The pharmacy dispensed the drug based on the assigned number. All physicians and pharmacists were blinded to the product number and the patient's group.

### 3.2. Inclusion Criteria

Inclusion criteria included patients with preeclampsia, defined as SBP > 140 or DBP > 90 after 20 weeks of gestation associated with proteinuria (either mild or with severe features of preeclampsia), aged between 22 and 40 years, full-term (> 37 weeks of gestation), and classified as American Society of Anesthesiologists (ASA) I or II, undergoing elective cesarean delivery. Exclusion criteria comprised patients with obesity (body mass index, BMI > 35 kg/m2), gestational age < 37 weeks, active labor, early labor, ruptured membranes, or a history of previous cesarean deliveries, diabetes, or gestational diabetes.

We included patients diagnosed with mild or severe preeclampsia. Mild preeclampsia was defined as the presence of hypertension (blood pressure > 140/90) without evidence of any organ damage in the patient, while severe preeclampsia was characterized by end-organ damage. Severe features of preeclampsia included SBP > 160, DBP > 110, thrombocytopenia, impaired liver function with twice the normal concentration of liver enzymes, right upper quadrant pain, progressive renal insufficiency with creatinine > 1.1 mg/dl, pulmonary edema, and new onset of cerebral or visual abnormalities.

### 3.3. Spinal Anesthesia

After obtaining informed consent, a cesarean section was performed following the spinal injection of either ropivacaine or bupivacaine. In all patients, 500 cc of IV fluid was prescribed before conducting spinal anesthesia. The first group (n = 45) received ropivacaine, and the second group (n = 45) received bupivacaine. The procedure was carried out at the level of L3 - 4 by an expert anesthesiologist using a 25 G Quincke spinal needle. The spinal anesthesia was performed by a single anesthesiologist to ensure consistency and reliability.

An isobaric dose of 0.5% ropivacaine or 0.5% bupivacaine was injected through the spinal needle. The ropivacaine dose was 15 mg of a 0.5% solution, and the bupivacaine dose was also 15 mg of a 0.5% solution. The level of anesthesia after the spinal injection in both groups was measured and was up to T4 in all patients.

### 3.4. Measurements

Demographic variables, including maternal age, gestational age, parity, gravidity, BMI, and co-morbidities, were recorded. Routine monitoring was continuously performed, incorporating ECG, non-invasive BP, and pulse oximetry (SPO_2_).

Baseline hemodynamic parameters, including heart rate and systolic and diastolic blood pressure, were recorded before intrathecal injection. The patient's blood pressure was measured and recorded at the baseline before spinal anesthesia and then every 2 minutes for 6 minutes after the intrathecal injection of local anesthetic. After delivery, blood pressure was measured every 5 minutes in the first 30 minutes. Bradycardia was treated with atropine (0.5 mg for heart rates less than 60), and hypotension and bradycardia were treated with 5 mg ephedrine intravenously. In addition to SBP, DBP, and PR, the ephedrine dose and pain score after surgery were also recorded.

Hypotension was defined as a > 20% decrease in MAP below the baseline in both groups during the period from the induction of spinal anesthesia to delivery (12); a 5 mg bolus of ephedrine was administered to restore the MAP at or above 80% of the baseline within 60 seconds (13).

To determine the pain score, a numerical rating scale (NRS) was used, with 0 indicating no pain and 10 representing the most severe pain as rated by the patient. For the time to return of movement in recovery, knee movement (flexion) was used as the target point. The duration of time from spinal anesthesia injection up to knee movement was used as a marker for the return of the motor block.

### 3.5. Statistical Analysis

Continuous data were presented as mean and standard deviation (SD), while categorical data were expressed as No (%). The normality of quantitative data was assessed using the Kolmogorov-Smirnov test of normality. For normally distributed data, a *t*-test was employed for the statistical analysis of two groups. A nonparametric Mann-Whitney U-test was used for skewed or ordered categorical data in the analysis of 2 groups. The chi-square test (%) was utilized for comparisons involving categorical data. Time-related variables of scores were analyzed using the Wilcoxon signed-rank test, and for normally distributed data, ANOVA followed by post hoc multiple comparison tests were performed. Additionally, Cohen's d test was used to measure and compare the difference between the amount of decrease in SBP and DBP in the 2 groups. All statistical tests were 2-sided and conducted at a significance level of F = 0.05. The analysis was carried out using IBM SPSS v. 22 Statistics.

## 4. Results

The mean age of all patients was 31.40 ± 8.53 years old. Age, height, gravidity, and parity were not significantly different between the 2 groups (P > 0.05) (Table 1). The Kolmogorov-Smirnov test indicated that distributions for age (P = 0.245), first SBP (P = 0.506), DBP (P = 0.327), and first pulse rate (P = 0.666) were not significantly different between the 2 groups, and the distribution of variables was normal.

**Table1.** Demographic Variables in Ropivacaine and Bupivacaine Groups

Variables	Ropivacaine (n = 45), No (%)	Bupivacaine (n = 45), No (%)	P-Value
**Age**	31.37 ± 8.8	31.42 ± 9.04	0.980
**Gravidity**			0.905
Null gravidity	2 (4.4)	2	
1 gravidity	17 (37.8)	15 (33.3)	
1 < gravidity	26 (57.8)	28 (62.2)	
**Parity**			0.136
No parity	16 (35.6)	22 (48.9)	
1 parity	9 (20)	12 (26.7)	
1 < parity	20 (44.4)	11 (24.4)	

**Figure 1.**
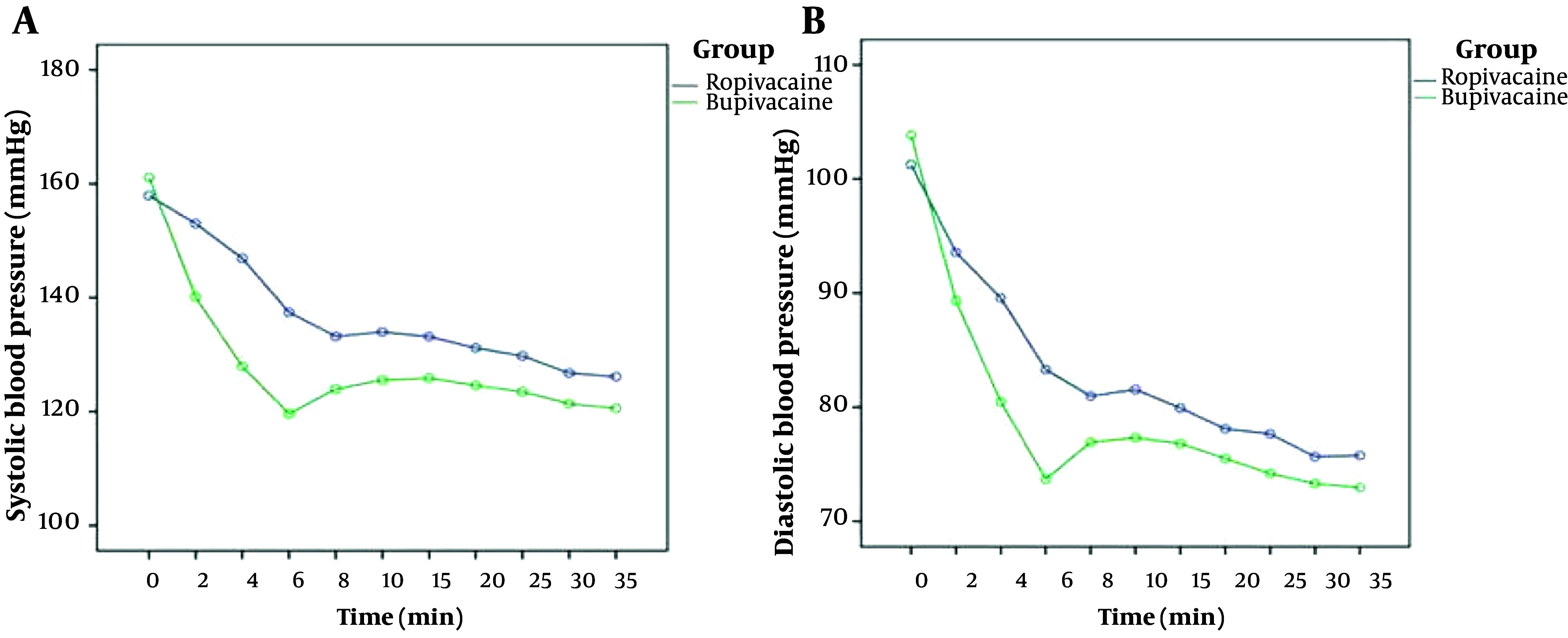
The trend of systolic blood pressure (SBP) and DBP at various time points after spinal anesthesia in Ropivacaine and Bupivacaine (Marcain) group.

At the time of pre-spinal injection of anesthesia, SBP showed no significant difference between the two groups (P = 0.341). Systolic blood pressure (SBP) significantly decreased in both groups after the spinal injection of anesthetic (P = 0.017) compared to pre-spinal injection. The mean SBP was significantly lower in the bupivacaine group compared to the ropivacaine group at all measured time points in the study (2, 4, 6, 8, 10, 15, 20, 25, 30, and 35 minutes) (Figure 1) (P < 0.05).

Pre-anesthesia diastolic blood pressure (DBP) was not significantly different between the 2groups (P = 0.48). DBP significantly decreased after spinal injection compared to pre-anesthesia in both groups (P < 0.001) (Figure 1). DBP was significantly higher at 4 and 6 minutes after spinal anesthesia in the ropivacaine group compared to the bupivacaine group (P < 0.001 and P = 0.005, respectively). At other time points, DBP was not significantly different between the 2 study groups (P > 0.05).

**Figure 2.**
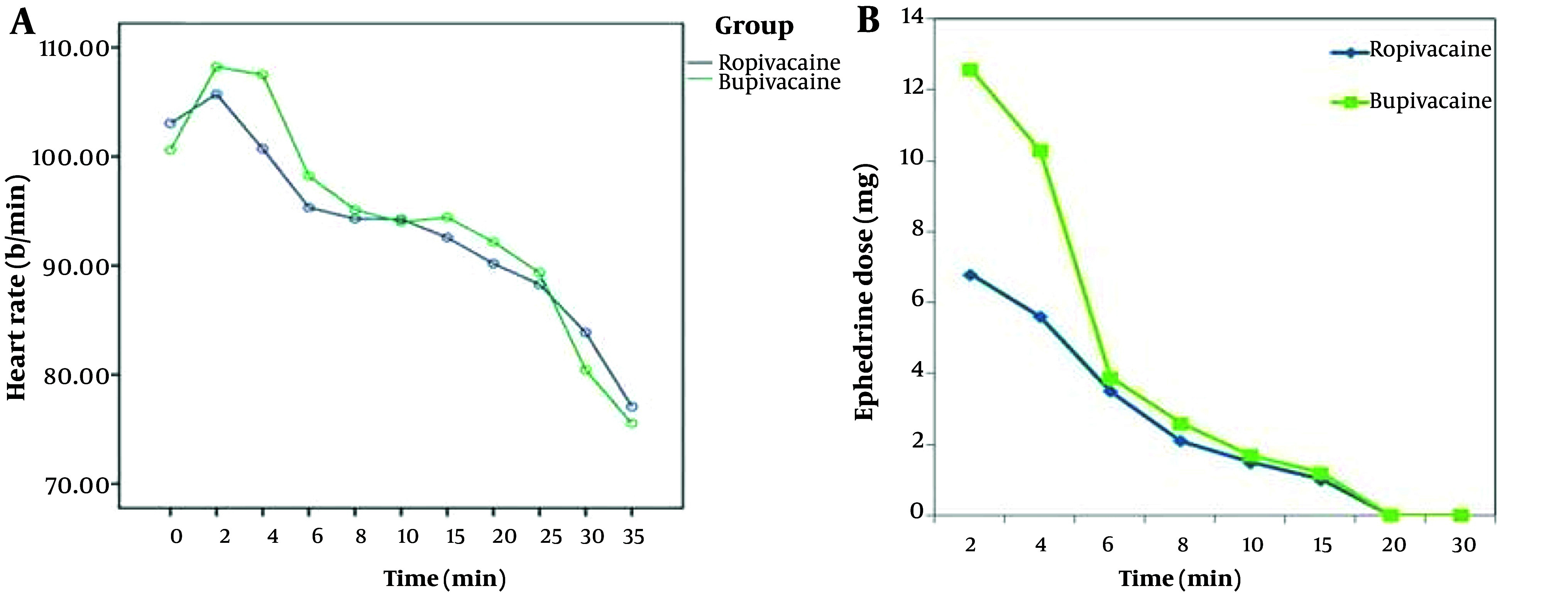
The trend of heart rate (h) and ephedrine dose at various time points after spinal anesthesia in Ropivacaine and Bupivacaine (Marcain) group.

The mean pre-anesthesia heart rate (HR) was not significantly different between the two groups (P = 0.26). The mean HR was not significantly different between the two groups at any time point (P < 0.05) (Figure 2). 

At 2 minutes after spinal anesthesia, SBP, DBP, and HR showed the most significant decrease in both groups and we compared the amount of decrease in means in both groups (Cohen's d test). The amount of decrease in SBP at 2 minutes after spinal injection in ropivacaine (d = 0.327) was significantly less than in the bupivacaine (d = 1.145) group. Similarly, the decrease in DBP at 2 minutes after spinal injection in the ropivacaine (d = 0.649) group was significantly less than in the bupivacaine (d = 3.037) group. The decrease in HR at 2 minutes after spinal injection in ropivacaine (d = 0.190) was significantly less than in the bupivacaine (d = 0.601) group.

The amount of ephedrine used after spinal anesthesia was significantly different at 2 and 4 minutes in the ropivacaine group compared to the bupivacaine group (P = 0.012, P = 0.025). It was not significantly different at other time points after intrathecal anesthesia injection (P > 0.05) (Figure 2). 

Interestingly, the post-operative pain score at 1 hour in recovery was not significantly different between the ropivacaine and bupivacaine groups (P > 0.05). The time to move the knee was also significantly lower in the ropivacaine group compared to the bupivacaine group (P < 0.001) (Table 2). 

**Table 2.** Post-operative Pain Score at 1 Hour in Recovery

Variables	Ropivacaine (n = 45)	Bupivacaine (n = 45)	P-Value
**Pain score in recovery**	1.22 ± 1.21	1.70 ± 1.59	0.15
**Return of movement**	20.00 ± 13.18	40.11 ± 11.28	< 0.001

## 5. Discussion

Spinal anesthesia is the safest method for cesarean section in preeclampsia due to severe airway complications. However, hypotension poses a major challenge for anesthesiologists during this procedure. Spinal anesthesia blocks sympathetic effects on the vascular system (14). The induced sympathectomy results in vasodilation in both arteries and veins, leading to a subsequent decline in systemic vascular resistance (SVR) and hypotension (15). Hemodynamic perturbation is a concern for both the anesthesiologist and the mother and fetus, which becomes apparent after spinal anesthesia, particularly in cases involving ropivacaine.

Anesthesiologists have explored various variables to reduce spinal-induced hypotension. It is believed that the occurrence of spinal anesthesia-induced hypotension is associated with the local anesthetic dose. By using bupivacaine or ropivacaine with a lower anesthetic dose, the incidence of hypotension can be significantly reduced.

However, even with a lower bupivacaine dose (15 mg), hypotension could still occur. Ropivacaine has been suggested by some researchers as a substitute for bupivacaine in spinal anesthesia for cesarean sections (16, 17). We used hyperbaric ropivacaine, which produces less motor and sympathetic block than isobaric or hypobaric ropivacaine (18) and has a shorter duration of motor block compared to bupivacaine (19). However, ropivacaine has not been used for spinal anesthesia in preeclampsia patients. In our study, we demonstrated that 15 mg of ropivacaine produced a parallel and effective clinical profile with a shorter duration of sensory and motor block compared with 15 mg of hyperbaric bupivacaine 0.5% for elective cesarean section. This advantage is of paramount importance in these patients.

Due to sympathetic overflow in preeclamptic parturients and subsequent block, their drop in blood pressure could be more dose-dependent. If a lower dose of anesthetics is used, patients with preeclampsia experience less hypotension during spinal anesthesia for cesarean section than healthy parturients (20). This underscores our study findings, as a lower dose of ropivacaine was superior to bupivacaine in maintaining blood pressure.

Previous results showed that low-dose bupivacaine spinal anesthesia is associated with a lower risk of hypotension than previously believed, and it can, therefore, be safely used in preeclamptic women undergoing cesarean delivery (21). However, a lower dose of bupivacaine may not be appropriate for cesarean section analgesia. Later studies showed that ropivacaine has an advantage with less influence on hemodynamics in cesarean delivery (22).

Spinal anesthesia for cesarean delivery with ropivacaine in women with preeclampsia is associated with modest hemodynamic changes of no clinical significance, as shown in Zhao et al.'s study (23). Gunduz et al.'s study demonstrated that both ropivacaine and bupivacaine provide equivalent labor analgesia with high maternal satisfaction, and no adverse obstetric or neonatal outcomes were observed in either group (24). Others have recommended ropivacaine not only for its minimal impact on hemodynamics but also for its shorter duration of sensory and motor block, leading to faster recovery in cesarean section (25).

Another important aspect of our study was the movement in recovery after spinal anesthesia. Parturients have a hypercoagulable state, which could increase the risk of venous thromboembolism after cesarean section if patients remain non-ambulatory. Movement recovery was faster in the ropivacaine group compared to the bupivacaine group. This implies that ropivacaine is a better choice for preeclampsia patients, as early ambulation is critical to decreasing the risk of deep vein thrombosis (DVT). The post-operative pain score at 1 hour in recovery was not significantly different between the ropivacaine and bupivacaine groups. This suggests that both drugs are equally effective in managing post-operative pain during the recovery period. However, it is important to note that this was accompanied by a delayed motor return in the bupivacaine group.

### 5.1. Conclusions

In conclusion, ropivacaine could be a preferable choice compared to bupivacaine for spinal anesthesia during cesarean sections in patients with preeclampsia. This preference is attributed to its ability to induce less spinal hypotension, provide better hemodynamic control, require less ephedrine usage, and facilitate faster ambulation of the patient. Further investigation is warranted to gain a deeper understanding of the mechanisms underlying the decreased incidence of hypotension with ropivacaine compared to bupivacaine.

## Data Availability

The dataset presented in the study is available on request from the corresponding author during submission or after publication. The data are not publicly available due to confidentiality.

## Clinical Trial Registration Code:

IRCT20150317021497N6.
